# Open questions: some unresolved issues in biodiversity

**DOI:** 10.1186/1741-7007-11-118

**Published:** 2013-12-06

**Authors:** Anne E Magurran

**Affiliations:** 1 Centre for Biological Diversity, School of Biology, University of St Andrews, St Andrews, Fife, KY16 8LB, Scotland, UK

## 

Biodiversity - or biological diversity - is a term now so familiar that politicians use it to persuade us that they really care about the natural world. Many papers, and indeed entire journals, are devoted to explaining how species coexist, why some localities are more diverse than others, and to tracking the rate at which biodiversity is being lost. It might seem that there is little to add to the debate. Yet, while there has been remarkable progress in understanding how ecological communities are structured, and how best to protect them, there are still many unresolved issues. Here are just three of them.

## Relative abundance of species

It must always have been obvious to observers that species vary in their abundances. Darwin remarked on the ‘beautiful diversity and proportion of kinds’ in his *Origin of Species*. We know that all ecological communities are characterized by a few common and typically many rare species. Although this pattern is ubiquitous, ecologists still struggle to provide a convincing explanation why species abundances are as uneven as they are. Dozens of models have been proposed (the first in 1932). Some of these take account of biological interactions such as competition, while others reflect the statistical behavior of large numbers. Since models can be based on conflicting assumptions yet generate patterns seen in the real world, a good ‘fit’ to empirical data does not in itself vindicate the theory underpinning the model. There is still a need for a much better understanding of the processes that influence the relative abundance of species, and that determine which species are abundant and which ones are rare. This will likely take account of species traits, and the positioning of a species in its range (populations tend to be larger when taxa are close to the center of their range). But the spatial and temporal context of a community is also important as species turnover in space and time plays a crucial role in maintaining the diversity of an assemblage. Understanding the processes that shape diversity over both macroecological and local assemblage scales will play an essential part in this [1].

## Anthropogenic change and biodiversity

Many diversity measures have been developed with the goal of finding a metric that can quantify the effects of impacts such as pollution on natural communities. This quest has been only partially successful. One reason is that community responses to different types of disturbance can be complex [2]. Moreover, even the best known predictive framework, the Intermediate Disturbance Hypothesis, has been subject to growing criticism [3]. A further problem is that a diversity metric reported in isolation is uninformative. It is only when there are good comptive data on species identities and abundances in an undisturbed community that meaningful conclusions can be drawn.

## Biodiversity offsetting

Biodiversity offsetting is increasingly hailed as a way of balancing the demands of development and conservation. It provides compensation for damaged sites. For example, if habitat such as woodland is destroyed to make way for housing or industry, offsetting means that equivalent habitat must be created or restored to protect the same level of biodiversity. In principle this sounds encouraging, but there are considerable challenges even when developers are keen to abide by the spirit as well as the letter of local guidelines. For example, some ancient habitats are irreplaceable, and it may be difficult or impossible to recreate ecological communities that have been established over decades and form part of important ecological networks. The Lawton report [4] discusses many of the concerns that biodiversity offsetting raises, and makes recommendations for dealing with them. In short, given the knowledge gaps that exist in relation to the processes that shape biodiversity, and how natural systems respond to impacts, biodiversity offsetting is not an easy solution, and substantial uncertainties remain.
